# Supplementary material for: Molecular Investigation of Klebsiella pneumoniae from Clinical Companion Animals in Beijing, China, 2017–2019
Source: Pathogens. 2021 Feb 27;10(3):271. doi: 10.3390/pathogens10030271 (PMC7997213; doi:10.3390/pathogens10030271)
Supplement: Supplementary file 1 [file pathogens-10-00271-s001.zip › Supplementary Files/Supplementary Materials.docx]

**Supplementary information of “Molecular Investigation of *Klebsiella pneumoniae* from clinical companion animals in Beijing, China, 2017-2019”**

**Supplementary Figure**


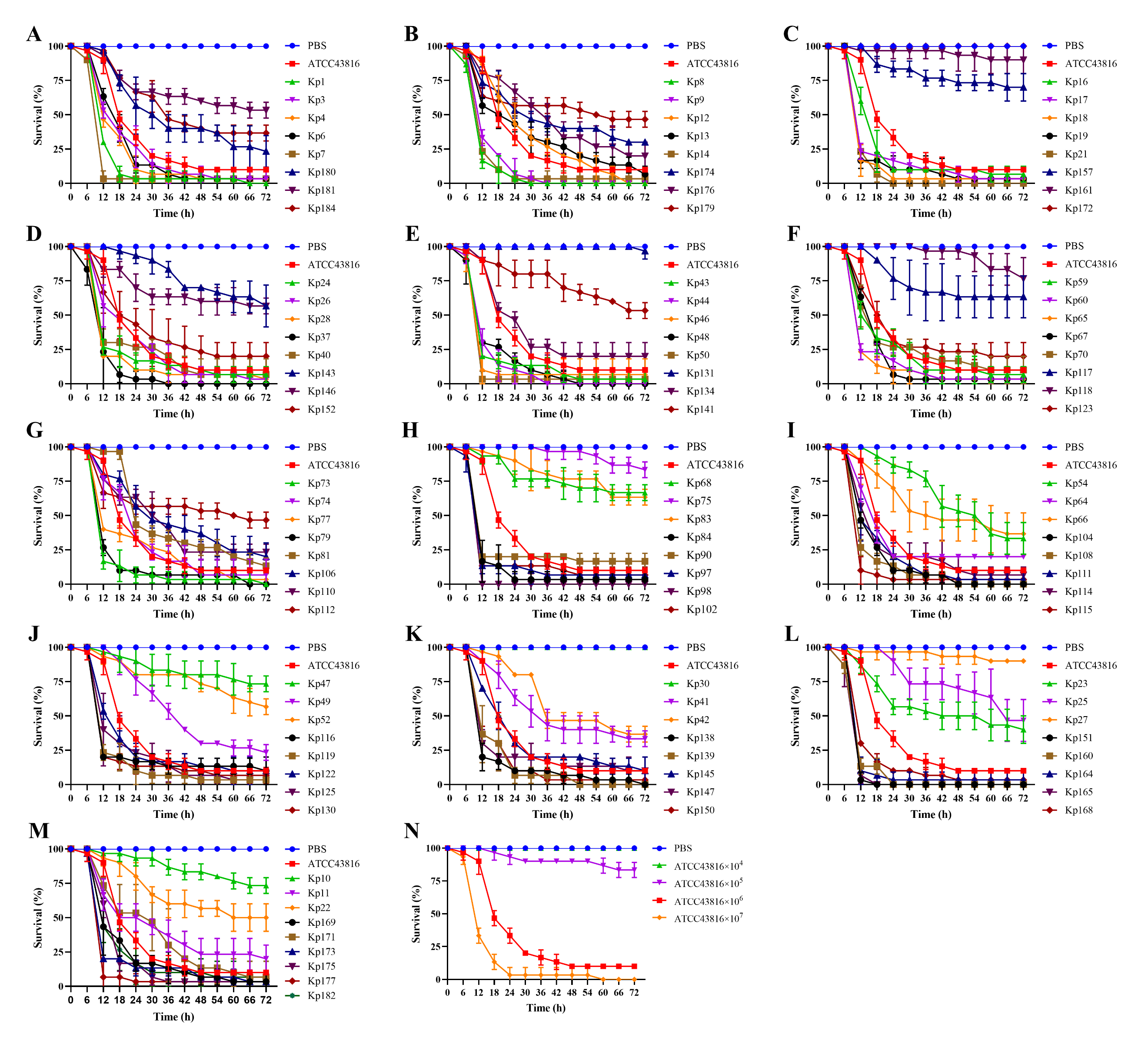


**Figure S1.** *K. pneumoniae* infection of *Galleria mellonella* lethality. (A-M) Larvae injected with PBS or 10^6^ CFU of 105 *K. pneumoniae* isolates from companion animals, and survival was monitored over 72h after infection. (N) Mortality of larvae infected with PBS or ATCC43816 (10^4^, 10^5^, 10^6^, 10^7^ CFU) was dose-dependent, and survival was monitored over 72 h after infection. PBS and ATCC43816 are the negative and positive control groups, respectively.

**Supplementary Table**

**Table S1.** MIC (μg/mL) of antimicrobial agents for clinical *K. pneumoniae* isolates from companion animals.

| Antimicrobial agent | All isolates (n=105) |  | Dog (n=85) | | |  | Cat (n=20) | | | *p*-value‡ |
| --- | --- | --- | --- | --- | --- | --- | --- | --- | --- | --- |
|  | Resistance, % |  | MIC_50_ | MIC_90_ | Resistance, % |  | MIC_50_ | MIC_90_ | Resistance, % |  |
| Amoxicillin-clavulanate | 78 (74.3) |  | 32/16 | >256/128 | 60 (70.6) |  | 64/32 | >128/64 | 18 (90.0) | 0.074 |
| Piperacillin-tazobactam | 32 (30.5) |  | 8/4 | >256/4 | 23 (27.1) |  | 8/4 | >256/4 | 9 (45.0) | 0.117 |
| Ceftazidime-avibactam | 5 (4.8) |  | 0.25/4 | 1/4 | 3 (3.5) |  | 0.25/4 | 1/4 | 2 (10.0) | 0.241 |
| Cefotaxime | 48 (45.7) |  | 0.125 | >256 | 34 (40.0) |  | 256 | >256 | 14 (70.0) | 0.015* |
| Cefepime | 40 (38.1) |  | 0.06 | >256 | 28 (32.9) |  | 32 | >256 | 12 (60.0) | 0.025* |
| Meropenem | 4 (3.8) |  | 0.06 | 0.125 | 2 (2.4) |  | 0.06 | 0.125 | 2 (10.0) | 0.163 |
| Imipenem | 5 (4.8) |  | 0.5 | 1 | 3 (3.5) |  | 0.5 | 1 | 2 (10.0) | 0.241 |
| Aztreonam | 43 (41.0) |  | 0.5 | >256 | 31 (36.5) |  | 64 | >256 | 12 (60.0) | 0.054 |
| Ciprofloxacin | 47 (44.8) |  | 0.25 | 128 | 32 (37.6) |  | 8 | 256 | 15 (75.0) | 0.003** |
| Enrofloxacin | 44 (41.9) |  | 0.5 | 64 | 32 (37.6) |  | 16 | 64 | 12 (60.0) | 0.068 |
| Gentamicin | 45 (42.9) |  | 0.5 | >256 | 33 (38.8) |  | 32 | >256 | 12 (60.0) | 0.085 |
| Amikacin | 23 (21.9) |  | 1 | >256 | 16 (18.8) |  | 2 | >256 | 7 (35.0) | 0.137 |
| Doxycycline | 55 (52.4) |  | 8 | 64 | 41 (48.2) |  | 64 | 64 | 14 (70.0) | 0.080 |
| Colistin | 8 (7.6) |  | 2 | 2 | 4 (4.7) |  | 2 | 16 | 4 (20.0) | 0.041* |
| Florfenicol | 52 (49.5) |  | 8 | >256 | 39 (45.9) |  | 16 | >256 | 13 (65.0) | 0.124 |
| Trimethoprim-sulfamethoxazole | 60 (57.1) |  | 4/76 | >32/608 | 44 (51.8) |  | >32/608 | >32/608 | 16 (80.0) | 0.022* |

‡ *p*-values were determined by Chi-square (χ^2^) and Fisher’s exact test in SPSS Statistics. **p* < 0.05, ***p* < 0.01.
