# Supplementary figures and images for: Molecular Investigation of Klebsiella pneumoniae from Clinical Companion Animals in Beijing, China, 2017–2019
Source: Pathogens. 2021 Feb 27;10(3):271. doi: 10.3390/pathogens10030271 (PMC7997213; doi:10.3390/pathogens10030271)

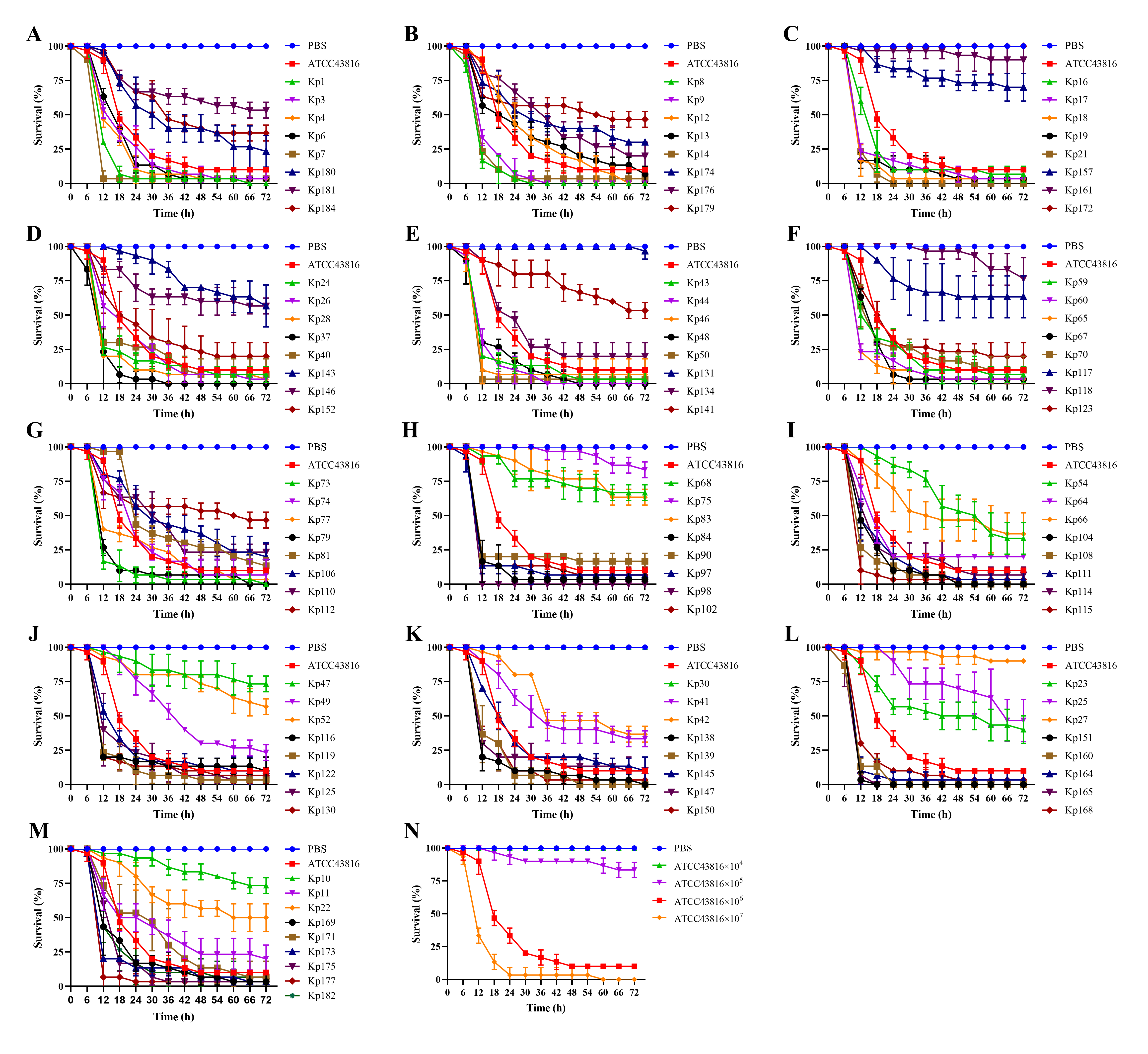

Supplement: Supplementary file 1 [file pathogens-10-00271-s001.zip › Supplementary Files/Figure S1.tif]
